# Impairment of early fracture healing by skeletal muscle trauma is restored by FK506

**DOI:** 10.1186/s12891-017-1617-y

**Published:** 2017-06-12

**Authors:** Brady J. Hurtgen, Beth E. P. Henderson, Catherine L. Ward, Stephen M. Goldman, Koyal Garg, Todd O. McKinley, Sarah M. Greising, Joseph C. Wenke, Benjamin T. Corona

**Affiliations:** 1 Extremity Trauma and Regenerative Medicine Task Area, US Army Institute of Surgical Research, 3698 Chambers Pass, BHT1, Fort Sam Houston, TX 78234 USA; 2 Department of Orthopaedic Surgery, Indiana University School of Medicine, Indianapolis, IN USA

**Keywords:** Adaptive immune response, Composite injury, Inflammation, Immunomodulation, Musculoskeletal, Open fracture, Orthopaedic trauma, Skeletal muscle injury, Volumetric muscle loss, Tacrolimus

## Abstract

**Background:**

Heightened local inflammation due to muscle trauma or disease is associated with impaired bone regeneration.

**Methods:**

We hypothesized that FK506, an FDA approved immunomodulatory compound with neurotrophic and osteogenic effects, will rescue the early phase of fracture healing which is impaired by concomitant muscle trauma in male (~4 months old) Lewis rats. FK506 (1 mg/kg; i.p.) or saline was administered systemically for 14 days after an endogenously healing tibia osteotomy was created and fixed with an intermedullary pin, and the overlying tibialis anterior (TA) muscle was either left uninjured or incurred volumetric muscle loss injury (6 mm full thickness biopsy from middle third of the muscle).

**Results:**

The salient observations of this study were that 1) concomitant TA muscle trauma impaired recovery of tibia mechanical properties 28 days post-injury, 2) FK506 administration rescued the recovery of tibia mechanical properties in the presence of concomitant TA muscle trauma but did not augment mechanical recovery of an isolated osteotomy (no muscle trauma), 3) T lymphocytes and macrophage presence within the traumatized musculature were heightened by trauma and attenuated by FK506 3 days post-injury, and 4) T lymphocyte but not macrophage presence within the fracture callus were attenuated by FK506 at 14 days post-injury. FK506 did not improve TA muscle isometric torque production

**Conclusion:**

Collectively, these findings support the administration of FK506 to ameliorate healing of fractures with severe muscle trauma comorbidity. The results suggest one potential mechanism of action is a reduction in local T lymphocytes within the injured musculoskeletal tissue, though other mechanisms to include direct osteogenic effects of FK506 require further investigation.

**Electronic supplementary material:**

The online version of this article (doi:10.1186/s12891-017-1617-y) contains supplementary material, which is available to authorized users.

## Background

Approximately 5 – 50% of open fractures present either delayed or non-union depending on injury severity and location, and patient characteristics [1, 2]. The concomitant loss of surrounding muscle tissue has been associated with poor healing outcomes in open fracture patients [2, 3] and experimentally confirmed in animal models [4, 5]. There are likely multiple mechanisms by which severe trauma to the surrounding musculature may impair fracture healing, to include impaired revascularization, loss of muscle-derived stem cells that support bone regeneration, and alteration of the local milieu of muscle-derived growth factors and myokines [6, 7]. However, heightened and prolonged immune responses are inherent to trauma [8] and are a principle mediator of musculoskeletal healing. It is therefore plausible that severe muscle trauma compounds the local immune response and thereby impairs fracture healing.

We recently described the recruitment of CD4^+^ (helper) and CD8^+^ (cytotoxic) T lymphocytes to both damaged muscle and fracture callus following complex musculoskeletal injury [4]. The recruitment of T lymphocytes was amplified through 28 days post-injury in comparison to a simple fracture and was associated with impaired healing [4]. CD4^+^and CD8^+^ T lymphocytes are key components of the adaptive immune system and have been shown to regulate musculoskeletal tissue degeneration and repair [9, 10]. However, depletion studies also implicate lymphocytes as deleterious to fracture repair [11]. For instance, CD4^+^ T cells can negatively influence the receptor activator of NF-kB-ligand/receptor activator of NF-kB/osteoprotegerin (RANKL/RANK/OPG) osteoclastogenesis pathway by activating stromal cells via cytokines and CD40-CD40L interactions and by activating bone destructing osteoclasts through the production of cytokines (RANKL and IL-17) [12]. CD8^+^ T cells may also negatively influence fracture healing [13], however exact mechanisms are not well defined. Altogether, the activation, polarization, differentiation, and magnitude of specific T cell functions are influenced by the complexity of injury and may be both beneficial and detrimental for musculoskeletal regeneration.

Therapeutically targeting T lymphocytes is an attractive strategy to restore fracture healing following severe musculoskeletal trauma. A viable tool is FK506 (Tacrolimus) because it both directly and indirectly inhibits T cell mediated immune responses [14]. Tacrolimus is an FDA approved macrolide that inhibits IL-2 dependent T cell activation by inhibiting enzymatic activity of calcineurin and subsequent translocation of transcription factor nuclear factor of activated T cells (NFAT) [14]. FK506 is also effective in reducing gene expression of pro-inflammatory, but not anti-inflammatory cytokines [14] and therefore potentially promotes T helper 2 (Th2) versus T helper 1 (Th1) responses. Moreover, FK506 has been shown to directly mediate osteogenic differentiation of mesenchymal stem cells in vitro [15], and therefore may augment challenged fracture healing in vivo [16]. FK506 also has neurotrophic properties that may improve functional recovery of the traumatized musculature [17]. That being said, the treatment of simple fractures or rhBMP-2 treated segmental defects with systemic administration of FK506 did not augment fracture healing [16, 18]. However, it remains plausible that fracture healing impaired by compounding inflammation secondary to concomitant muscle trauma (i.e., open fracture) [4] may be rescued by FK506.

In the current study, we primarily tested the hypothesis that systemic FK506 administration improves the recovery of mechanical strength of fractured tibia when accompanied by comorbid muscle trauma using an established rat model of open fracture [4]. As a putative mechanism, the effect of FK506 administration on the innate and adaptive immune responses in the injured bone and muscle were evaluated.

## Methods

### Animals

All protocols and animal care guidelines were approved (A14-033) by the Institutional Animal Care and Use Committee at the United States Army Institute of Surgical Research. All components were conducted in compliance with the Animal Welfare Act, the Implementing Animal Welfare Regulations and in accordance with the principles of the Guide for the Care and Use of Laboratory Animals. Inbred male Lewis rats (350-400 g; ~4 months of age) were purchased from Harlan Laboratories and housed in a specific pathogen-free animal facility. All rats received a pre-surgical (~30 min prior) administration of buprenorphine-SR (1.2 mg/kg; s.c.) for pain management and a post-surgical x-ray was taken to ensure proper fracture fixation. All animals were euthanized while heavily sedated, by injection of Fatal Plus (sodium pentobarbital, 150 mg/kg; intracardiac).

### Experimental design

Volumetric muscle loss (VML) injury has previously been shown to impair endogenous healing of rat tibia non-segmental osteotomy (OST) [4] and BMP-2 mediated healing of a segmental defect (SD) in the rat tibia [19] and femur [5]. The current work was designed to determine if the immunomodulatory compound, FK506, restores the early phase of recovery of tibia mechanical properties during endogenous bone regeneration of impaired fracture healing.

**Fig. 1:**
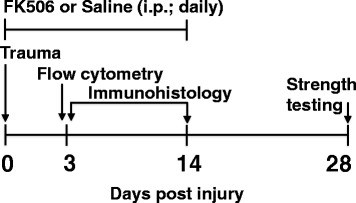
Experimental time course. The schematic depicts the therapeutic regimen (FK506 or saline) and experimental assays carried out over the course of 28 days post OST + VML. Trauma consisting of OST + VML was generated at day 0. FK506 or saline control was administered daily by the intraperitoneal route from day 0 to day 14 following injury. At 3 days post-injury, flow cytometry and immunohistochemical analysis of damaged muscle and adjacent fracture region was carried out, immunohistochemical analysis was also analyzed at 14 days post-injury. At 28 days post-trauma strength testing assays of bone and muscle were carried out

Male Lewis rats underwent surgical creation of an endogenously healing tibia osteotomy (OST) with or without concomitant ipsilateral tibialis anterior (TA) muscle VML injury (OST + VML). Briefly, the OST was performed approximately 5 mm proximal to the tibia-fibula junction and stabilized with a 1.25 mm Kirschner pin inserted in the medullary cavity from the tibial plateau [4]. VML injury was surgically created in the middle third of the adjacent TA muscle using a punch biopsy (6 mm), as we have reported previously [20, 21]. X-rays were acquired with 3 days post-injury to ensure adequate tibia stabilization. Injured rats were allotted randomly to systemic saline or FK506 treatment groups. FK506 (1 mg/kg; Sigma-Aldrich) was administered in sterile saline by daily injection (i.p.) beginning immediately after surgery and for 14 days post-injury, during the primary period post-injury when the musculoskeletal immune response is exacerbated in this model [4]. This dosage of FK506 has previously been shown to induce a therapeutic concentration of FK506 systemically [18]. Rats were followed out to 28 days post-injury, at which time musculoskeletal physiological and mechanical assessments were performed (*n* = 6-8/group; Statistical power (1-ß) was approximately 0.7). Previous reports using this and similar models have indicated that at this time post-injury fracture healing is not fully resolved, permitting observations of an impaired rate of healing by concomitant VML injury [4, 22, 23]. To determine if FK506 attenuated the cellular immune response in OST + VML, muscles samples were harvested at 3 days post-injury for flow cytometric analysis (*n* = 4/group). Additionally, tibiae were collected at 3 and 14 days post-injury, in a follow-on analysis for quantitative immunohistological analyses (*n* = 3/group; Fig. 1).

### Bone mechanical testing

Three-point bending was performed on a set of rat tibiae at 28 days post-injury, using methods previously described [4]. Load-deflection curves were used to obtain maximum load and stiffness.

### Muscle functional assessment

In vivo functional testing of TA muscles was performed at 28 days post-injury using methodology previously described [20, 21, 24]. Briefly, TA muscle in vivo physiological properties were measured in anesthetized rats (isoflurane 1.5 – 2.0%) using a dual-mode muscle lever system (Aurora Scientific, Inc., Mod. 305b). Subcutaneous needle electrodes were inserted in the posterior compartment of the lower limb on each side of the common peroneal nerve. Optimal voltage (2 – 5 V) was set with a series of tetanic contractions (5 – 10 contractions; 150 Hz, 0.1 ms pulse width, 400 ms train). Then, a skin incision was made at the antero-lateral aspect of the ankle and the distal tendons of the extensor digitorum longus and extensor hallicus longus muscles was isolated and severed above the retinaculum [20]. TA muscle maximal isometric tetanic torque was measured (150 Hz) with the ankle at a right angle.

### Cellular isolation from muscle tissue

Cells were isolated from the middle third of the TA muscle that encompassed the muscle defect by enzymatic digestion as previously described [4]. Briefly, the defect was surgically isolated and the mass was determined. Tissue was incubated with collagenase type II and dispase for 90 min at 37 °C. Cells were further released by gentle mechanical disruption and filtered through a 70 μm cell strainer. Erythrocytes were lysed with ammonium-chloride-potassium lysing buffer and cells were filtered through a 40 μm cell strainer washed, and resuspended in PBS containing 0.5% FBS and 0.1% sodium azide. Viable cells were quantified using trypan blue exclusion and a hemocytometer.

### Quantification of cellular infiltrates in muscle defect by flow cytometry

Muscle-derived cells were labeled with fluorochrome-conjugated antibodies and analyzed by flow cytometry as previously described [4]. Cells were incubated with anti-CD32 antibody to block Fc receptors and labeled with either cocktail monoclonal antibodies to identify macrophages or T lymphocytes. The macrophage (MØ) cell cocktail included anti-CD45 (clone OX-1), anti-CD11b (WT.5), anti-CD68 (ED1), anti-CD86 (24F), and anti-CD163 (ED2). The T lymphocyte cell cocktail consisted of anti-CD45, anti-CD3 (IF4), anti-CD4 (OX-35), and anti-CD8α (OX-8). Labeled cells were fixed with 1% paraformaldehyde and enumerated by fluorescence-activated cells sorting using a MACSQuant flow cytometer (Miltenyi Biotec). Data were analyzed using MACSQuantify software (Miltenyi Biotec). Gating strategies to identify cellular populations included the following: CD45^+^ hematopoietic cells, CD45^+^CD11b^+^CD68^+^ MØ, CD45^+^CD11b^+^CD68^+^CD86^+^ M1-like MØ, CD45^+^CD11b^+^CD68^+^CD163^+^ M2-like MØ, CD45^+^CD3^+^CD4^+^ T helper and CD45^+^CD3^+^CD8α^+^ T cytotoxic lymphocytes. Cell numbers of each gated population were determined by multiplying the percentage of cells by the total number of viable cells recovered from the respective defect. All cell numbers were normalized per gram of excised muscle tissue.

### Histology

Tibiae were fixed in 10% neutral buffered formalin and decalcified in a formic acid bone decalcifier (Immunocal, Decal Chemical Corp, Tallman NY) using similar methodology as previously described [4]. Samples were processed in paraffin and embedded in a longitudinal orientation. Tibiae were cut in 8 μm sections and deparafinized. Immunohistochemical (CD4, BioRad: MCA55GA, 1:100; CD8a, BD Pharmingen: 550,298, 1:100; CD68, BioRad: MCA341R, 1:200) staining was performed using standard methodology.

Bright field images were acquired with a Zeiss Axio Scan.Z1 and stitched into a large composite image. Composite images were saved separately as a 24-bit, 96 dpi color images. All images were analyzed in MetaMorph (Molecular Devices LLC., Sunnyvale CA). For display purposes only, images were produced in Keynote by down-converting, without introducing any changes in brightness or contrast. With each inflammatory marker (CD4, CD8, and CD68) the region of the callus between the bone and on the anterior aspect of the tibia was selected for analysis, care was taken to not select mineralized bone or skeletal muscle fibers (Additional file 1). The image was color thresholded (hue, saturation, and intensity) to identify individual inflammatory markers (by saturation threshold). The percentage of area covered was determined off of the area of the total selected region and were analyzed using binary images. Analyses were conducted by an investigator blinded to treatment group; in the event that multiple serial sections from a single tibia stained for the same inflammatory marker the percent area from each section was averaged and used for analysis (*n* = 3 rats/group/time/marker). Tibiae sections used as negative controls were used to verify analysis of inflammatory markers.

### Statistics

Dependent variables were analyzed using one- or two-way ANOVA or Student’s t-test. In the event of a significant ANOVA, Fisher’s post-hoc testing was performed. Statistical significance was achieved at alpha of 0.05. Statistical testing was performed using Prism 6 for Mac OSX (Graphpad Inc). Data is presented as box (25 to 75 percentile with median line) and whisker (minimum and maximum response) plots.

## Results

### Systemic FK506 administration improves healing of fracture with concomitant VML injury but not recovery of muscle strength

**Fig. 2:**
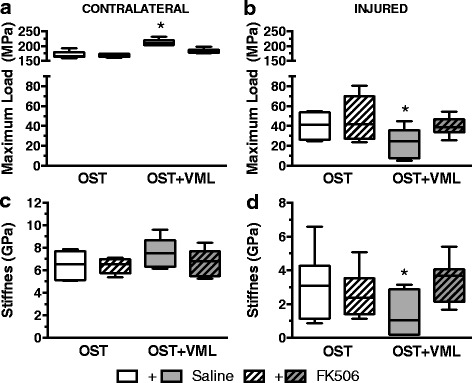
Mechanical testing of bone following FK506 treatment of OST + VML injury. Three-point bending mechanical testing of (**a** & **c**) contralateral and (**b** & **d**) injured tibiae was performed at 28 days post-injury. * ≠ OST-saline & OST + VML-FK506; *p* < 0.05. Group responses are presented as box (25 to 75 percentile with median line) and whisker (minimum and maximum response) plots; *n* = 6-8/group

Previously, the immunomodulatory drug FK506 did not improve healing of a simple femur OST without concomitant muscle trauma [18]. Herein, FK506 delivery also failed to improve healing of an isolated tibia OST, evidenced by similar mechanical properties of injured OST-only tibiae from saline or FK506-treated rats 28 days post-injury (Fig. 2a & b). However, often fracture occurs with a concomitant loss of surrounding musculature (e.g., Gustilo-Anderson type II and III open fracture), which can impair bone regeneration [4]. In a model of this clinical scenario, VML injury significantly reduced (OST + VML-saline) tibia mechanical strength (−41%; *p* = 0.028) and stiffness (−54%; *p* = 0.039) compared to OST-only groups 28 days post-injury, with 5 of 6 observations in the OST + VML-saline group having mechanical property values lesser than the median response of OST-saline (Fig. 2). Coinciding with diminished mechanical strength in the injured limb, the contralateral tibia mechanical strength was significantly increased in OST + VML treated with saline, compared to all other groups (*p* < 0.001). Notably, FK506 restored tibia mechanical properties in OST + VML rats to OST-only levels (Fig. 2b).

**Fig. 3:**
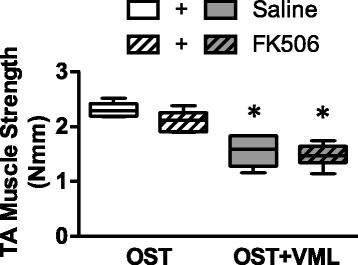
TA muscle strength assessment after FK506 therapy. In vivo peak isometric tetanic torque was assessed at 28 days post-injury. *each OST + VML group < respective OST group, *p* < 0.05. Group responses are presented as box (25 to 75 percentile with median line) and whisker (minimum and maximum response) plots; *n* = 6-8/group

The effect of FK506 administration on in vivo isometric TA muscle strength was also assessed 28 days post-injury (Fig. 3). In OST-only groups, TA muscle strength was similar between saline and FK506-treated rats (*p* = 0.112). The surgical creation of VML injury (OST + VML-saline) resulted in a 33% decrease in neural-evoked isometric torque compared to OST-saline (*p* < 0.001), and FK506 administration did not ameliorate TA muscle strength deficits (OST + VML-saline vs. –FK506: *p* = 0.492).

### Systemic FK506 administration modulates the immune response within VML injured muscle

**Fig. 4:**
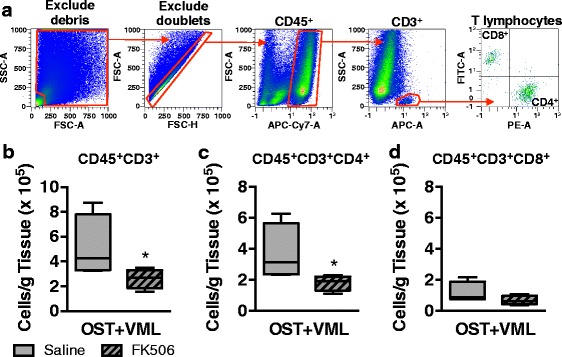
Flow cytometric analysis of T lymphocytes infiltrating skeletal muscle after OST + VML treated with FK506. TA muscles were isolated from OST + VML groups treated with saline or FK506 at 3 days post-injury to evaluate immune cell infiltration by flow cytometry. The middle third region of the TA muscle was collected, digested, and cellular content was isolated. **a** Events were gated (red polygons) to eliminate debris and doublets, and cells were then gated for CD45^+^CD3^+^ T lymphocytes, CD45^+^CD3^+^CD8^−^CD4^+^ T helper lymphocytes, and CD45^+^CD3^+^CD4^−^CD8^+^ cytotoxic T lymphocytes. **b**-**d** Data for each cell population are normalized per gram of muscle tissue and presented as box (25 to 75 percentile with median line) and whisker (minimum and maximum response) plots; *n* = 4/group. *p < 0.05

VML injury induces a heightened and prolonged local pro-inflammatory immune response within the traumatized musculature that associates with impaired fracture healing [4]. To investigate the effect of systemic FK506 administration on the immune response to VML injury, first flow cytometric quantification of TA muscle infiltrating CD45^+^CD3^+^CD4^+^ (T helper) and CD45^+^CD3^+^CD8^+^ (T cytotoxic) lymphocytes was performed 3 days post-injury (Fig. 4a). In OST + VML groups, FK506 significantly reduced the number of CD45^+^CD3^+^CD4^+^ cells by 51% (*p* = 0.037). CD45^+^CD3^+^CD8^+^ cells were not significantly reduced by FK506 treatment (−43%; *p* = 0.087; Fig. 4b - d).

**Fig. 5:**
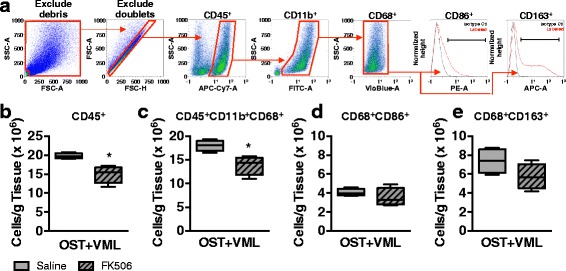
Flow cytometric analysis of macrophages infiltrating skeletal muscle after OST + VML treated with FK506. TA muscles were isolated from OST + VML groups treated with saline or FK506 at 3 days post-injury to evaluate immune cell infiltration by flow cytometry. The middle third region of the TA muscle was collected, digested, and cellular content isolated. **a** Events were gated (*red polygons*) to eliminate debris and doublets, and cells were then gated for CD45^+^ hematopoietic cells, CD45^+^CD11b^+^CD68^+^ macrophages, CD45^+^CD11b^+^CD68^+^CD86^+^ M1-like macrophages (*black bar*), and CD45^+^CD11b^+^CD68^+^CD163^+^ M2-like macrophages (*black bar*); **b**-**e** Data for each cell population are normalized per gram of muscle tissue and presented as *box* (25 to 75 percentile with median line) and whisker (minimum and maximum response) plots; *n* = 4/group. *p < 0.05

The effect of FK506 therapy on macrophage populations recruited to damaged muscle at 3 days post-injury was also investigated. Again, using flow cytometry we quantified muscle infiltrating CD45^+^ (hematopoietic), CD45^+^CD11b^+^CD68^+^ (MØ), CD45^+^CD11b^+^CD68^+^CD86^+^ (M1-like MØ), and CD45^+^CD11b^+^CD68^+^CD163^+^ (M2-like MØ) cells (Fig. 5a). FK506 reduced CD45^+^ hematopoietic cells by 24% (*p* = 0.008) and CD68^+^ macrophages by 22% (*p* = 0.013), but had no significant effect on M1 and M2 polarization or recruitment in comparison to saline treated animals (Fig. 5b - e).

### Fracture site infiltration of T lymphocytes but not macrophages is attenuated by systemic FK506

**Fig. 6:**
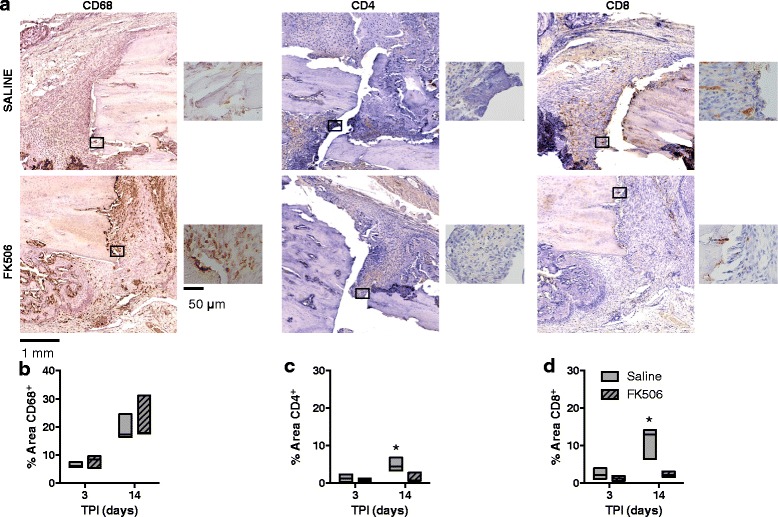
Evaluation of immune cells infiltrating fracture callus of OST + VML treated with FK506 using immunohistological staining. Injured tibiae were collected at 3 and 14 days post-injury from OST + VML injured groups that received saline or FK506 treatment. Tissue was sectioned and immunohistochemical analysis was carried out to detect CD68^+^ macrophages, CD4^+^ T cells, and CD8^+^ cytotoxic T cells, using horseradish peroxidase for detection. **a** Representative images from the fracture callus site for each treatment group at the 14 day time point is presented, with magnified inserts presented adjacent to image and identified by a box in the primary image. Scale bars = 1 mm and 50 μm, respectively. **b**-**d** The relative area of the fracture callus populated by CD68^+^, CD4^+^, and CD8^+^ cells were quantified. Group responses are presented as floating box plots (minimum to maximum with median line); *n* = 3/group. * saline >3 day saline & 14 day FK506; *p* < 0.05. TPI, time post-injury

As a potential link between heightened muscle inflammation and impaired fracture healing, it has been observed that conditions of heightened local and systemic inflammation correspondingly protract fracture site inflammation and disrupt bone regeneration [4, 25]. To further establish this relationship, the presence of CD4^+^ and CD8^+^ T cells and CD68^+^ macrophages was quantified within the fracture callus at 3 and 14 days post-injury in saline or FK506-treated rats with OST + VML injury (Fig. 6). No differences in macrophage or lymphocyte markers were observed between groups during the early phase of repair at 3 days post-injury. At 14 days post-injury, the fractional area of CD68^+^ expression was elevated in saline and FK506-treated rats compared to 3 days post-injury, but again no difference in macrophage marker presence was observed. The fractional area of both CD4^+^ and CD8^+^ expression was significantly elevated compared to 3 days post-injury in OST + VML treated with saline, which was significantly attenuated in FK506 treated rats (OST + VML-saline vs. -FK506: CD4^+^ *p* = 0.008; CD8^+^ *p* = 0.001).

## Discussion

The primary finding of this study is that the FDA approved immunomodulatory drug, FK506 (Tacrolimus), improves the early recovery of tibia mechanical properties following osteotomy with concomitant severe muscle trauma (i.e., VML injury). Notably in this endogenously healing fracture model [4] and others that require rhBMP-2 treatment to recover [5, 19], VML injury impairs fracture healing and therefore the improvement in mechanical strength mediated by FK506 delivery reflects a restoration of the rate of endogenous fracture healing. These findings support the exploration of immunomodulatory therapies for augmentation of impaired fracture healing secondary to a loss of surrounding muscle tissue (e.g., type II and IIIa tibia open fracture).

In polytrauma and myopathy, heightened inflammation has been coupled with either poor fracture healing or poor bone health [25–29]. In each of these conditions, immunomodulation (i.e., macrophage or complement inhibition) has effectively improved bone healing [25, 30]. Similarly, under conditions of exacerbated inflammation, such as implantation of xenogeneic demineralized bone grafts in the abdominal musculature [31] or genetically modified xenogeneic muscle implants into femur segmental defect [16], FK506 has improved bone formation. However, previous work has shown that FK506 does not improve endogenous or rhBMP-2 mediated fracture healing without extensive muscle trauma [16, 18], which is likely because fractures without large muscle trauma do not result in excessive local immune responses [4]. The current findings agree with Voggenreiter et al. [18], as FK506 did not enhance healing of an osteotomy without VML injury, but go on to demonstrate that FK506 re-establishes the early recovery of mechanical properties in the presence of concomitant VML injury, which elicits exacerbated inflammation. In association with improved tibia mechanical properties, FK506 administration reduced the acute infiltration of T cells and macrophages in VML-injured musculature and T cells in the fractured bone within the first 2 weeks post-injury. These data therefore support further testing of the broad working hypotheses that: 1) VML injury prompts a heightened local immune response that disrupts fracture healing and 2) that an immunomodulatory therapy (e.g., FK506) normalizes the musculoskeletal immune response, and thereby rescues fracture healing.

While FK506 ameliorated the recovery of tibia mechanical properties, treatment did not improve neuromuscular strength after VML injury. Muscle strength deficits after VML injuries have been primarily attributed to the traumatic loss of contractile tissue [32, 33]. However, strength deficits are at least partially compounded by acute intramuscular nerve damage and it is suspected that the heightened immune response to VML injury causes secondary muscle damage. Given the well-known neurotrophic effects of FK506 [17, 34], it was therefore anticipated that FK506 administration would improve functional recovery of the remaining muscle mass. The systemic dose (1 mg/kg) used in this study was adequate to attenuate macrophage and lymphocyte infiltration within VML injured muscle, however, this dose has conflicting results regarding peripheral nerve regeneration [35, 36]. Collectively, these findings indicate that while attenuation of the heightened local immune response caused by VML injury may be beneficial to fracture healing, continued efforts are required to improve skeletal muscle regeneration and recovery of strength.

Improvement of fracture healing by FK506 was consistently associated with attenuated T lymphocyte infiltration within the traumatized muscle and bone. Given that T lymphocytes have been shown to drive the development and function of osteoclasts through the production of the pro-inflammatory cytokine IL-17 and RANKL [37], FK506 mediated attenuation of this degenerative interaction may explain the improved fracture healing. The restorative effects of FK506 on fracture healing may also occur through modulation of multiple types of immune cells. Several reports suggest FK506 modulates innate immune cells to generate reduced responses to innate stimuli [38–40]. For example, dendritic cells and macrophages pretreated with FK506 were shown to produce lower amounts of pro-inflammatory cytokines after exposure to lipopolysaccharide, a pathogen associated molecular pattern (PAMP) [38, 39]. PAMPs and damage-associated molecular patterns (DAMPs) both signal through Toll-like receptor (TLR) pathways that are damped in TLR-ligand stimulated peripheral blood mononuclear cells isolated from patients who received FK506 [40]. FK506 also affects the regulatory T (Treg) cell population, which is known to dampen pro-inflammatory immune responses [41]. It is clear that the entire T cell population is not detrimental to fracture repair but instead individual T cell subsets uniquely regulate or impair the fracture repair process [13, 42–46]. Given the myriad of cellular targets upon which FK506 may interact and general IL-2-mediated suppression of T lymphocytes by FK506, further study is required to assess whether such a broad therapeutic effect is necessary to restore fracture healing after severe musculoskeletal trauma.

We were surprised by the differential response of macrophages in the bone and muscle tissue. A previous report of *mdx* mouse pathology demonstrated a coordinated macrophage response between diseased muscle and adjacent fracture bone that corresponded with impaired fracture healing [25]. However, FK506 reduced CD45^+^ cells, which were primarily (>90%) comprised of CD68^+^ macrophages, in the muscle 3 days post-injury while cell numbers of this population were unchanged in the fracture site at 3 or 14 days post-injury. It is possible that the reduction of macrophages in the muscle could lead to overall reduced pro-inflammatory cytokine/chemokine levels that impact downstream wound healing events in muscle and bone. Alternatively, the activation levels of macrophages at the fracture may be reduced to allow for improved wound healing despite the cell numbers being unaltered by FK506. Further investigation is required to conclusively determine the impact of the macrophage response on fracture healing following complex musculoskeletal trauma.

It has been established that local musculature provides vascular, paracrine, mechanical, and cellular support to adjacent bone [6, 7]. The partial loss of surrounding skeletal muscle in similar rodent open fracture models indicate that associated impairment of fracture healing is not necessarily driven by deficient revascularization of the fracture [22, 23], highlighting a potential deficiency in the activity of myokines [47–49] and muscle derived stem cells [50, 51] in the support of fracture healing under these conditions. Interesting interplay between immune responses and IGF-1 signaling [52] and muscle stem cells [53] support the idea that that while controlled inflammation is beneficial to muscle-bone communication, exacerbated inflammation following severe soft tissue trauma may restrict muscle derived support of osseous regeneration and thus contribute to impaired fracture healing.

FK506 may also directly influence osteoblast and osteoclast activity [54–58] and thereby improve fracture healing. Notably, FK506 has also been shown to promote osteogenic differentiation of mesenchymal stem cells via BMP receptor signaling in vitro [15]*,* highlighting the potential utility of this compound as an osteoinductive agent. In vivo FK506 had no effect on the quantity but improved the quality of bone formation in xenogeneic muscle graft-treated femur defect in athymic rats, potentially reflecting direct osteoblast/clast augmentation [25]. In relation to fracture healing, it is interesting that in animal models with a competent immune system, FK506 does not appear to augment endogenous or rhBMP-2 mediated bone regeneration when comorbid muscle trauma is not explicitly introduced, see Fig. 2 and [16, 18]. These findings suggest that in vivo the direct osteogenic effects of FK506 are more influential under conditions of heightened inflammation. Suffice to say that while impaired bone regeneration following open fracture can be driven by the dysregulated immune response provoked by VML injury [4], rescued fracture healing by FK506 may not be strictly related to immunomodulation.

There remain numerous questions regarding the use of FK506 or other immunomodulatory compounds for open fracture that are not addressed in this work. Notably, it is unclear whether FK506 is effective if delivered in a delayed manner, which is the likely clinical scenario. Relatedly, it is unclear if FK506 can be delivered either locally or systemically at an effective dose for musculoskeletal healing without potentiating infection. While these salient limitations are important to clinical translation, this initial study highlights the utility of FK506 in improving regeneration following complex musculoskeletal trauma.

## Conclusions

In summary, the present study offers initial evidence that reduction of the dysregulated immune response triggered by complex musculoskeletal injury with FK506, an FDA-approved drug (Tacrolimus), may play a role in rescuing impaired fractured healing; however, direct osteoinductive activity of the compound may also influence early fracture healing. In agreement with previous lymphocyte depletion studies [11], herein the data associate heightened T cell but not macrophage recruitment to the fracture site with impaired recovery of mechanical properties early during the fracture healing process. The current study also suggests that a mere attenuation of T cell infiltration within injured bone and adjacent muscle associates with improved bone healing. The results of this study highlights the potential of immunomodulatory drugs to treat orthopaedic musculoskeletal trauma and supports previous findings that the net T cell response to complex musculoskeletal injury is dysfunctional for efficient fracture healing.

## Additional file

Additional file 1: Representative image presenting selection of region for immunohistochemistry analysis. Quantitative analysis of CD4, CD8, and CD68 was conducted off of the whole bone section (A) by selecting the region of the callus (B). In all instances the callus region was traced in the area between the bone ends on the anterior aspect of the tibia without selection of mineralized bone or skeletal muscle fibers. C) The selected representative image corresponds to the image in Fig. 6 for reference. (PDF 3305 kb)

## Abbreviations

DAMPs: Damage-associated molecular patterns
NFAT: Nuclear factor of activated T cells
OPG: Osteoprotegrin
OST: Osteotomy
PAMP: Pathogen associated molecular pattern
RANK: Receptor activator of NF-kB
RANKL: Receptor activator of NF-kB-ligand
SD: Segmental defect
TA: Tibialis anterior
Th1: T helper 1
Th2: T helper 2
TLR: Toll-like receptor
Treg: Regulatory T cell
VML: Volumetric muscle loss
