# Supplementary figures and images for: Impairment of early fracture healing by skeletal muscle trauma is restored by FK506
Source: BMC Musculoskelet Disord. 2017 Jun 12;18:253. doi: 10.1186/s12891-017-1617-y (PMC5469075; doi:10.1186/s12891-017-1617-y)

A

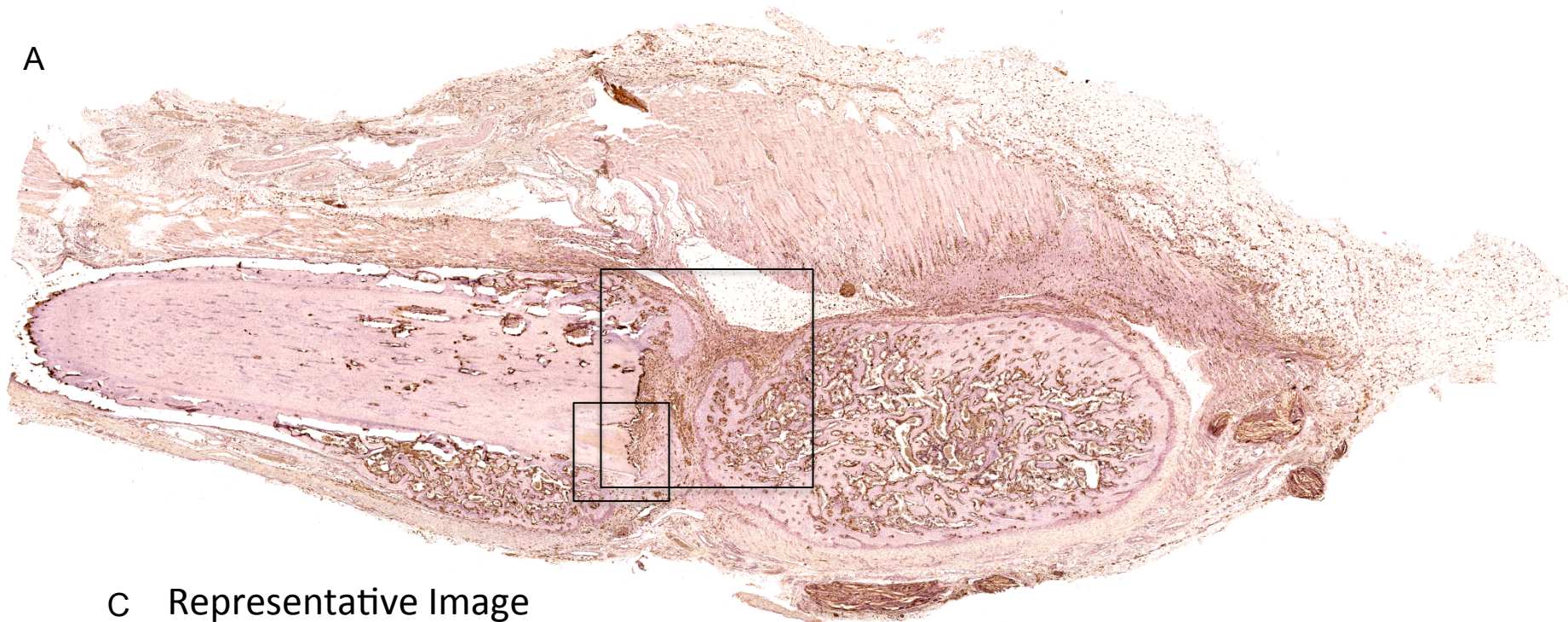

C Representative Image

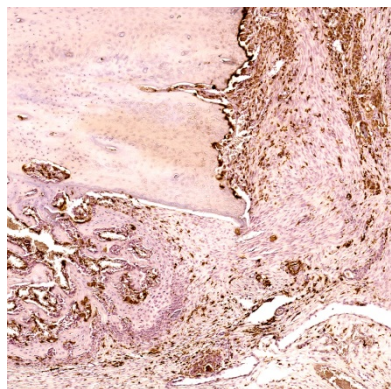

B

Selected Region

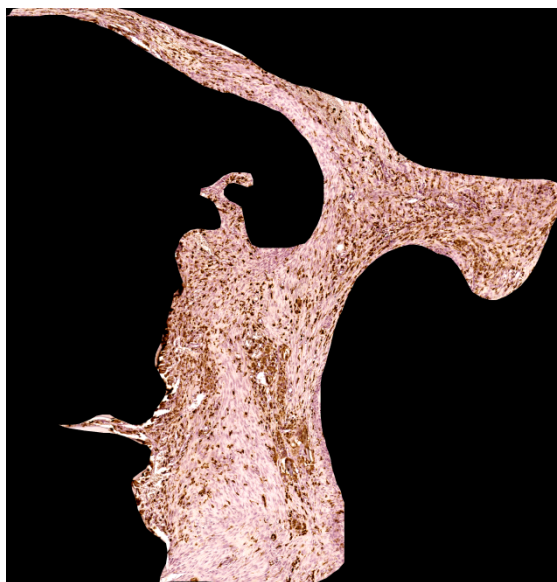

Supplement: Additional file 1: — Representative image presenting selection of region for immunohistochemistry analysis. Quantitative analysis of CD4, CD8, and CD68 was conducted off of the whole bone section (A) by selecting the region of the callus (B). In all instances the callus region was traced in the area between the bone ends on the anterior aspect of the tibia without selection of mineralized bone or skeletal muscle fibers. C) The selected representative image corresponds to the image in Fig. 6 for reference. (PDF 3305 kb) [file 12891_2017_1617_MOESM1_ESM.pdf]
